# Potent, multi-target serine protease inhibition achieved by a simplified β-sheet motif

**DOI:** 10.1371/journal.pone.0210842

**Published:** 2019-01-22

**Authors:** Xingchen Chen, Blake T. Riley, Simon J. de Veer, David E. Hoke, Jessica Van Haeften, Darren Leahy, Joakim E. Swedberg, Maria Brattsand, Perry J. Hartfield, Ashley M. Buckle, Jonathan M. Harris

**Affiliations:** 1 School of Biomedical Sciences, Queensland University of Technology, Brisbane, Queensland, Australia; 2 Department of Biochemistry and Molecular Biology, Biomedicine Discovery Institute, Monash University, Clayton, Victoria, Australia; 3 Institute for Molecular Bioscience, The University of Queensland, Brisbane, Queensland, Australia; 4 Department of Medical Biosciences, Umeå University, Umeå, Sweden; Uniwersytet Gdanski, POLAND

## Abstract

Engagement of an extended β-sheet is a common substrate/inhibitor interaction at the active site of serine proteases and is an important feature of Laskowski mechanism inhibitors that present a substrate-like loop to a target protease. This loop is cleaved but subsequently relegated forming a stable inhibitor/protease complex. Laskowski inhibitors are ubiquitous in nature and are used extensively in serine protease inhibitor design. However, most studies concentrate on introducing new sidechain interactions rather than the direct contributions of the substrate-like β-sheet to enzyme inhibition. Here we report the crystal structure of an simplified β-sheet inhibitory motif within the Sunflower Trypsin Inhibitor (SFTI) in complex with trypsin. We show that the intramolecular hydrogen bond network of this SFTI variant (SFTI-TCTR) engages the inhibitor sidechains that would normally interact with a target protease, giving mainchain interactions a more prominent role in complex formation. Despite having reduced sidechain interactions, this SFTI variant is remarkably potent and inhibits a diverse range of serine proteases. Crystal structural analysis and molecular modelling of SFTI-TCTR complexes again indicates an interface dominated by β–sheet interactions, highlighting the importance of this motif and the adaptability of SFTI as a scaffold for inhibitor design.

## Introduction

Serine proteases account for almost one-third of all proteases and occupy pivotal positions in controlling biochemical processes as diverse as digestion, homeostasis, signal transduction, immune responses and apoptosis [1, 2]. There is a sharp delineation between the digestive enzymes trypsin and chymotrypsin, which have comparatively relaxed substrate selectivity and proteases involved in the specialised processes whose fine control necessitates more restrictive substrate cleavage. Indeed, the irreversible nature of proteolytic cleavage together with proteolytic selectivity allows these enzymes to occupy a unique niche in cellular pathways where they act as unidirectional switches. Thus, proteases can facilitate steps of commitment in many signalling networks and afford attractive points for therapeutic inhibition. This paradigm has driven a series of successful campaigns to design serine protease inhibitors yielding a number of drugs that are in clinical use (see [3] for review).

The majority of therapeutic serine protease inhibitors are designed to be highly specific to minimise off-target effects. In contrast, many naturally occurring endogenous inhibitors of proteases show a relaxed specificity for their targets. An example of this is afforded by Lympho-epithelial Kazal-type-related inhibitor (LEKTI; encoded by the *SPINK5* gene) [4]. This multidomain inhibitor blocks the activity of at least 5 different serine proteases and has a profound effect on proteolytic activity in the stratum corneum. The loss or deficiency of LEKTI activity is responsible for Netherton syndrome, a severe and sometimes fatal skin disorder that has been shown to involve unrestrained activity from the kallikrein-related peptidases (KLKs) KLK5, KLK7 and KLK14 [5–8].

Although Netherton syndrome is comparatively rare, affecting 1 in 200,000 newborns, it is considered to be a model for many other chronic skin diseases. Accordingly, it is being vigorously targeted by pharmaceutical companies [9] as well as a number of academic laboratories [10]. As with other inhibitor design campaigns, much effort is being put into improving selectivity. Given the relaxed inhibitory specificity of LEKTI, it has been postulated that a synthetic, multiple-target inhibitor might be a useful replacement for treatment of diseases such as Netherton syndrome where LEKTI is deficient. However, this proposition is complicated by the fact that KLK5 and 14 are trypsin-like proteases cleaving C-terminal to arginine or lysine, whereas KLK7 is a chymotrypsin-like protease cleaving C-terminal to bulky aromatic and hydrophobic residues. LEKTI overcomes this challenge by virtue of its multiple Kazal domains having differential potencies and selectivity for a given protease.

Kazal domains are “standard mechanism” (Laskowski) inhibitors which mimic ideal protease substrates, presenting an exposed loop (known as the canonical loop) to a target protease [11]. This loop binds to the active site of proteases in a substrate-like manner and is cleaved at a scissile bond. However, unlike a typical protease substrate, Laskowski inhibitors maintain the cleaved termini of the reactive loop in a position which allows their religation [11]. Consequently, an equilibrium between cleavage and religation of the scissile bond occurs, resulting in the formation of a stable inhibitor/protease complex [11]. Previously we have designed potent and highly selective inhibitors using the canonical loop as it appears in Sunflower trypsin inhibitor-1 (SFTI-1) [12–14]. SFTI-1 is a cyclic, 14 amino acid peptide (GRCTKSIPPICFPD) stabilised by a dense intramolecular hydrogen bond network and a bisecting disulfide bond [15]. It is also one of the smallest natural inhibitors to conform to the Laskowski mechanism and is a potent inhibitor of trypsin (*K*_i_ = 0.1 nM) [15]. Mutation of just three residues corresponding to Schecter-Berger P1, P2 and P4 subsites [16] can redirect the inhibitory activity of SFTI-1 to a given protease, making it an ideal candidate for redesign and synthesis of variants [17]. To date, a number of proteases have been selectively inhibited by engineered SFTI inhibitors, including matriptase [18], KLK4 [12, 13], KLK5 [19], KLK7 [14, 20], KLK14 [19, 21], matriptase-2 [22] and the proteasome [23].

An important design consideration when engineering SFTI inhibitors is that there is a strong probability that the intramolecular hydrogen bond network will be disrupted. Previously, we customised the canonical loop of SFTI-1 to produce a potent and selective inhibitor of KLK4 [12] and in a subsequent study optimised the disrupted intramolecular hydrogen bond network to increase inhibitory potency by 125 fold [13]. We then extended this approach and produced a further version, SFTI-TCTR (GTCTRSIPPICNPN; substitutions are underlined). Design of this variant was solely focused on re-configuration of its internal hydrogen bond network rather than increasing the number of favourable interactions with a given target. We found that this variant exhibits potent inhibition towards trypsin (*K*_i_ = 0.7 nM), KLK5 (*K*_i_ = 2.0 nM), and KLK14 (*K*_i_ = 0.4 nM) [19], despite relatively fewer intermolecular interactions. Furthermore, SFTI-TCTR is also able to effectively inhibit KLK7 (*K*_i_ = 17 nM) [21] and thus can modulate all three of the pivotal kallikrein proteases in the epidermis. To explore the structural basis of this potent and broad range inhibitory activity, X-ray crystallographic analysis was performed on SFTI-TCTR in complex with trypsin. This data was then used as the basis for molecular dynamic simulation of SFTI-TCTR in complex with trypsin and KLK7.

## Materials and methods

### Peptide synthesis

All peptides inhibitors and substrates were synthesised on 2-chlorotrityl chloride resin (1.55 mmol Cl^-^/g, Iris Biotech) using an optimised solid phase peptide synthesis protocol for Fmoc chemistry [12, 24]. All Fmoc-protected amino acids were purchased from Iris Biotech. Peptide *para*-nitroanilide (pNA) substrates were synthesised on resin that had been prederivatised with 2 molar equivalents of para-phenylenediamine by overnight incubation with 5% diisopropylethylamine (DIPEA, Sigma-Aldrich) in dimethylformamide (DMF, Merk Millipore). SFTI inhibitors were synthesised as a linear peptide on 2-chlorotrityl resin using a Discover SPS Microwave Synthesiser (CEM Cooperation) followed by head-to-tail cyclisation as previously reported [19]. Chain elongation was achieved using 4 molar equivalents of Fmoc-protected amino acids, 1.1 molar equivalents of *O*-(6-Chlorobenzotriazol-1-yl)-N,N,N’,N’-tetramethyluronium hexafluorophosphate (HCTU, Chem-Impex) with 5% DIPEA in DMF as activator. 40% piperidine (Sigma-Aldrich) in DMF was used for N-terminal deprotection. Linear peptides were liberated from the resin by 0.5% TFA in dichloromethane (Sigma-Aldrich), precipitated by 10 volume of ice-cold diethyl ether and collected by centrifugation. For peptide-pNA substrates, oxidation of *para*-aminoanilide group was achieved by overnight incubation with 4 molar equivalents of oxone in a solution of acetonitrile:water (1:1 v/v). Microwave assisted head-to-tail cyclisation of SFTI inhibitors were carried out using equimolar DIPEA, HCTU and HOAt in DMF. Side chain protecting groups were removed by 2 hours incubation in a cleavage solution containing 95% TFA, 2.5% thioanisole, 1.25% triisopropylsilane and 1.25% H_2_O. Complete peptides were again pelleted using ice-cold diethyl ether and resolubilised in 10% isopropanol. Hydrophobic by-products were removed using C18 solid phase extraction cartridges (Grace Davison Discovery Sciences). Final purification was achieved by reverse-phase HPLC using a Jupiter Proteo 90 Å C18 column (Phenomenex). Peptides were eluted with a linear gradient of 10–90% isopropanol containing 0.1% TFA.

### X-ray crystallography

Bovine trypsin (Sigma, T8642) was dissolved in 50 mM MES buffer (pH 6.0) containing 50 mM benzamidine and 1mM CaCl_2_ to a final concentration of 20 mg/mL. 4 μL of protein solution was mixed with 4 μL of reservoir buffer (2.3 M (NH_4_)_2_SO_4_ and 0.1 M MES pH 6.0) and equilibrated over the reservoir buffer at room temperature. Benzamidine-inhibited trypsin crystals were washed and equilibrated in an inhibitor exchange buffer (0.1 M MES, pH 6.0, 2.5 M (NH_4_)_2_SO_4_ and 1 mM CaCl_2_) for 6 hours to remove benzamidine. Washed crystals were then transferred to fresh inhibitor exchange buffer supplemented with saturating amounts of SFTI-TCTR and soaked for a further 48 hours. Crystals were washed 3 times in 10 μL of fresh inhibitor exchange buffer to remove surface bound inhibitor and transferred into the same buffer supplemented with 20% (v/v) glycerol for cryoprotection before being flash-cooled to 100 K in a nitrogen stream. Crystals were irradiated using a Cu Kα rotating anode source at 45 kV and 30 mA, and diffraction data was collected at 100 K from a Rigaku R-Axis IV^++^ image plate. Indexing, scaling and merging of the data was performed by iMOSFLM [25] and Aimless [26]. All crystals were isomorphous with published SFTI-1/trypsin (PDB ID 1SFI) [15]. These coordinates were used as a starting structure for refinement with PHENIX [27] and Coot [28].

### PDB accession code

The atomic coordinates and structure factors of the trypsin-SFTI-TCTR complex reported here are deposited in the Protein Data Bank under code 6BVH.

### Protein expression, purification and active site titration

KLK7 was produced as a zymogen in the yeast *Pichia pastoris* strain X-33 previously developed and optimised by Dr Maria Brattsand [29]. Expressed proteins were purified using cation exchange chromatography on UnoSphere S (Biorad) and activated by treatment with enterokinase (10 units per milligram purified zymogen; Thermo Fisher Scientific) before a final polishing step to remove enterokinase, again using cation exchange chromatography. The active site concentration of purified KLK7 was determined using the serpin α1- proteinase inhibitor (A1PI) which binds to KLK7 with 1:1 stoichiometry. Briefly, a fixed amount of KLK7 was pre-incubated with a range of inhibitor concentrations at room temperature for 20 min before adding a constant concentration of substrate KHLY-pNA. The residual KLK7 activities were measured as the increase of absorbance at 405 nm for 10 min (ΔOD_405_/min) and plotted against respective inhibitor concentrations. The active site concentration was determined by linear regression to find the concentration of inhibitor required for complete KLK7 inhibition (ΔOD_405_/min drops to zero), which is equal to the concentration of KLK7 active site.

### Inhibition assays

Inhibitory property of SFTI inhibitors was assessed by determining the inhibition constant (*K*_i_) in competitive inhibition assays against a range of proteases as described before [19]. Serial dilutions of the inhibitor were pre-incubated with a fix concentration of protease in assay buffer (0.1 M Tris pH 8.0, 0.1 M NaCl and 0.05% Triton X-100) at room temperature for 10 min. Assays were initiated by addition of respective peptide-pNA substrates. Absorbance changes at 405 nm were monitored using a micro plate spectrophotometer (Bio-rad Benchmark Plus) over 5 min. Inhibition constants were calculated in GraphPad Prism 5.01 (La Jolla California, USA) by non-linear regression using the Morrison equation for tight binding inhibitors.

#### Molecular dynamic simulations

Molecular dynamic simulations were performed on the MonARCH and MASSIVE GPU clusters (Monash University). Initial atomic coordinates for the SFTI-TCTR/trypsin complex were obtained from the crystal structure described in this study. Coordinates for KLK7 were obtained from the PDB ID 2QXI [30], and coordinates for the SFTI-TCTR/KLK7 complex were created by merging the KLK7 and SFTI-TCTR/Trypsin structures, after alignment based on protease backbone atoms. Residue protonation states appropriate for pH 7.0 were assigned using PROPKA [31, 32]. Each protein was then placed in a rectangular box with a border of at least 12 Å of water on all sides of the protein, and the system charge was neutralized by addition of sodium or chloride counter-ions. Systems were parameterized using the AMBER ff14SB all-atom force field [33–35] in conjunction with the TIP3P explicit water model [36]. Systems were relaxed with 15000 steps of energy minimization, followed by equilibration. In equilibration, atoms’ initial velocities were randomly distributed according to a Maxwell-Boltzmann distribution at 100 K. Harmonic positional restraints of 100 kcal^-1^ mol^-1^ Å^-2^ were applied to protein backbone atoms and temperature was steadily increased from 100 K to 300 K over the course of 100 ps, with a Langevin damping coefficient of 5 ps^-1^. A Berendsen barostat [37] (τ_P_ = 0.1 ps) was then applied to equilibrate pressure to 1 atm and restraints were removed steadily over 200 ps. Production simulations were performed in the NPT ensemble without positional restraints, using an integration timestep of 2 fs, and saving snapshots every 5 ps for analysis. Three independent replicates of each system were simulated for 500 ns each. All simulations were performed using AMBER14 [38] with periodic boundary conditions, long-range interactions were computed using PME [39]. Root mean square deviation (RMSD) of Cα atoms for each frame in the trajectories was calculated after a least-squares fit on Cα to the initial frame. Hydrogen bonds are calculated using the Wernet-Nilsson cone criterion [40, 41]. Structural representations were produced using PyMOL version 1.8 [42] and VMD 1.9.2 [43], and all trajectory manipulation and analysis was performed with a combination of custom scripts, MDTraj [41], SciPy [44], Matplotlib [45], iPython [46] and VMD 1.9.2 [43].

## Results

### Crystal structure of SFTI-TCTR in complex with trypsin

Crystals of trypsin in complex with SFTI-TCTR were subject to X-ray crystallographic analysis (Table 1). The entire protease and inhibitor residues at the interaction surface were well defined in the electron density map, with the exception of Asn_12_−Asn_14_ of the inhibitor, indicating increased flexibility in this region.

**Table 1:** X-ray crystallographic data collection and refinement statistics for trypsin/SFTI-TCTR complex (PDB ID 6BVH).

Trypsin/SFTI-TCTR complex	
Wavelength (Å)	1.542
Resolution range (Å)	63.24−1.93 (1.97−1.93)
Space group	P 2_1_ 2_1_ 2_1_
Unit cell parameters	61.68, 63.24, 69.32, 90, 90, 90
Total reflections	73320 (4705)
Unique reflections	20647 (1331)
Multiplicity	3.6 (3.6)
Completeness (%)	97.9 (95.6)
Mean I/sigma(I)	27.1 (17.0)
Wilson B-factor	13.7
R-merge	0.035 (0.063)
R-meas	0.041 (0.075)
Reflections used for R-free	5.41% (4.98%)
R-work	0.132 (0.130)
R-free	0.158 (0.158)
Number of non-hydrogen atoms	2132
Macromolecules	1732
Ligands	40
Water	360
Protein residues	237
RMS (bonds)	0.007
RMS (angles)	0.83
Ramachandran favored (%)	98
Ramachandran allowed (%)	100
Ramachandran outliers (%)	0
Clashscore	1.14
Average B-factor	18.4
Macromolecules	14.9
Ligands	38.7
Solvent	33.1

Statistics for the highest-resolution shell are shown in parentheses.

**Fig 1:**
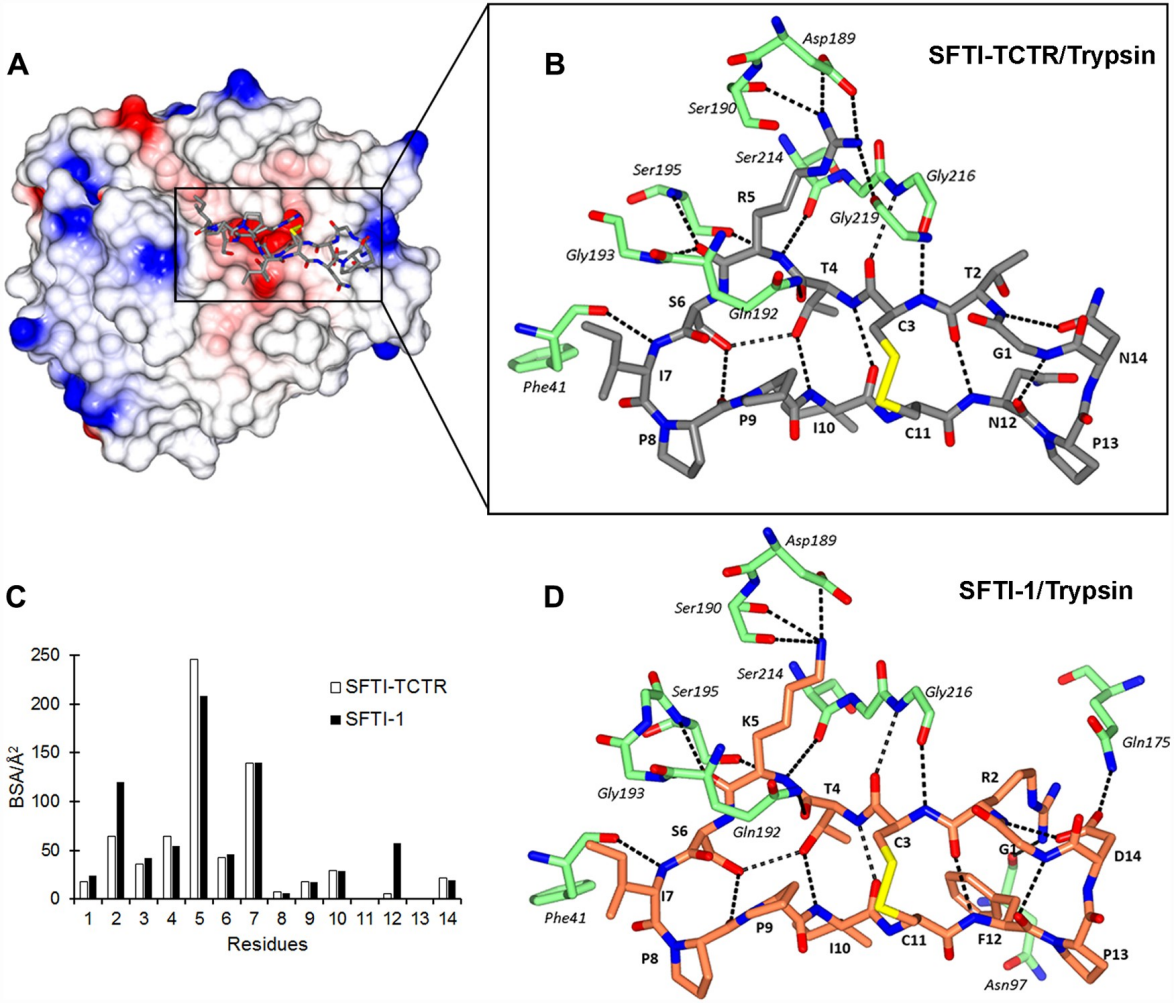
Crystal structure of SFTI-TCTR in complex with trypsin. (A) Overall structure of the complex. SFTI-TCTR (stick representation, carbon atoms grey) is located in the active site of trypsin (electrostatic potential surface representation). (B) Intra- and intermolecular hydrogen bonds of SFTI-TCTR when complexed with trypsin (stick representation, carbon atoms green). (C) Buried surface area (BSA) of SFTI-TCTR and SFTI-1 residues in the interface with trypsin. (D) Intra- and intermolecular hydrogen bonds SFTI-1 (stick representation, carbon atoms coral) when complexed with trypsin (PBD ID 1SFI). Hydrogen bonds are shown as black dashed lines. Structural representations were generated using CCP4MG [48].

The overall structure of the SFTI-TCTR/trypsin complex (Fig 1A) is similar to that reported for the SFTI-1/trypsin complex [47], indicated by an RMSD of 0.21 Å over the backbone atoms of the two structures. The hydrogen bonds between SFTI-TCTR and trypsin are largely confined to the P3-P1’ residues, as summarised in Fig 1B and Table 2. The sidechain of the P1 residue Arg_5_ extends into the S1 pocket of trypsin and interacts through an extensive hydrogen bond network and a salt bridge. A conserved short antiparallel β-sheet is formed across the inhibitor/protease interface, involving hydrogen-bonding pairs Arg_5_ N/Ser_214_ O, Cys_3_ N/Gly_216_ O and Cys_3_ O/Gly_216_ N. Meanwhile, SFTI-TCTR restores the original intramolecular hydrogen bond network seen in the wildtype SFTI-1, adopting an identical β-sheet motif.

**Table 2:** Intermolecular hydrogen bonds in the complexes of SFTI-TCTR/trypsin (PDB ID 6BVH) and SFTI-1/trypsin (PDB ID 1SFI).

Trypsin	SFTI-TCTR		SFTI-1	
Residue/atom	Residue/atom	Distance (Å)	Residue/atom	Distance (Å)
Asn_97_ O			Arg_2_ NH2	3.05
Gly_216_ O	Cys_3_ N	2.99	Cys_3_ N	3.08
Gly_216_ N	Cys_3_ O	3.18	Cys_3_ O	3.15
Gln_192_ NE2	Thr_4_ O	2.81	Thr_4_ O	2.99
Ser_195_ OG	Arg_5_ N	2.92	Lys_5_ N	2.89
Ser_214_ O	Arg_5_ N	3.03	Lys_5_ N	3.31
Asp_189_ OD1	Arg_5_ NH1	2.99	Lys_5_ NZ	3.19
Ser_190_ O			Lys_5_ NZ	3.11
Ser_190_ OG	Arg_5_ NH1	2.85	Lys_5_ NZ	2.99
Asp_189_ OD2	Arg_5_ NH2	2.77		
Gly_219_ O	Arg_5_ NH2	2.98		
Gly_193_ N	Arg_5_ O	2.68	Lys_5_ O	2.60
Ser_195_ N	Arg_5_ O	2.90	Lys_5_ O	3.06
Phe_41_ O	Ile_7_ N	2.98	Ile_7_ N	3.01
Gln_175_ NE2			Asp_14_ OD2	2.51

The surface area of SFTI-TCTR buried in the complex interface is 690.7 Å^2^, accounting for 47.7% of its total solvent accessible surface area, as analysed using the PISA server [49]. This is slightly lower than that of SFTI-1 (763.6 Å^2^ and 52.1% respectively). The difference is largely due to substitutions at inhibitor residues 2 and 12 (Fig 1C). In SFTI-TCTR, Arg_2_ is substituted by the non-contacting residue threonine. Phe_12_ and Asp_14_ are substituted by asparagine. These substitutions were introduced with the aim of reducing selective sidechain contacts while maintaining the intramolecular hydrogen bond network [19], which is consistent with the crystal structure. Compared to wildtype SFTI-1, the sidechains of Thr_2_, Asn_12_ and Asn_14_ are folded inwards, reducing the intermolecular interactions at these positions (Fig 1B and 1D). Analysis of the interface residues using the PISA server [49] shows that Asn_12_ (SFTI-TCTR) contributes 5.8 Å^2^ of buried surface area in the inhibitor/trypsin interface, accounting for only 7.42% of the residue’s total surface area, while Phe_12_ (SFTI-1) buried 57.3 Å^2^ (63.9%) of surface area in the bound state. This indicates that the bulky sidechain of Phe_12_ makes a better geometric fit with the relatively hydrophobic cleft framed by Asn_97_, Leu_99_ and Trp_215_ of trypsin, reflected by a buried surface area of 130.1 Å^2^ (54.7%) in these residues.

Overall, the comparison of crystal structures between SFTI-TCTR/trypsin and SFTI-1/trypsin shows that intramolecular hydrogen bonds are maintained in SFTI-TCTR to preserve an extended β-sheet structure with trypsin, while the interactions outside this β-sheet structure are largely minimised.

### SFTI-TCTR interacts with KLK7 primarily through its backbone atoms

Previously, SFTI-1 and SFTI-TCTR were assayed against a series of trypsin-like proteases and shown to be potent inhibitors of trypsin, KLK5 and KLK14, consistent with the inhibitor’s lysine and arginine P1 residues. In contrast to the trypsin-like kallikreins, KLK7 has a chymotrypsin-like specificity preferring phenylalanine or tyrosine at the P1 position of substrates [30]. As expected from this preference, KLK7 is poorly inhibited by SFTI-1 with *K*_i_ = 4800 nM (Table 3). However, SFTI-TCTR was an effective inhibitor of KLK7 with 286-fold increased potency (*K*_i_ = 17 nM) [21]. To gauge the effect of Arg_5_ at the P1 position in isolation from other substitutions, we also assessed inhibition of KLK7 by a SFTI variant where only Lys_5_ has been substituted by arginine (GRCTRSIPPICFPD, abbr. SFTI-RCTR). This variation increased inhibitory potency against KLK7 by 22 fold (*K*_i_ = 220 nM; Table 3), indicating that KLK7 has a strong preference for arginine over lysine at the P1 position.

**Table 3:** Inhibition of trypsin, KLK5, KLK7 and KLK14 by SFTI-1 and SFTI variants.

Inhibitor	Sequence	Enzyme	*K*_i_ (nM)	Substrate
SFTI-1	GRCT**K**SIPPICFPD	Trypsin	0.020 ± 0.002 [19]	Ac-YASR-pNA
		KLK7	4800 ± 200	KHLY-pNA
SFTI-RCTR	GRCT**R**SIPPICFPD	KLK7	220 ± 6	KHLY-pNA
SFTI-TCTR	GTCT**R**SIPPICNPN	Trypsin	0.70 ± 0.07 [19]	Ac-YASR-pNA
		KLK5	2.0 ± 0.1 [19]	Ac-YRSR-pNA
		KLK14	0.40 ± 0.02 [19]	Ac-YANR-pNA
		KLK7	17.0 ± 0.4 [21]	KHLY-pNA

In order to achieve a structural understanding of inhibitory activity of SFTI-TCTR for KLK7, a model of the SFTI-TCTR/KLK7 complex was built by superimposing the SFTI-TCTR peptide from the SFTI-TCTR/trypsin structure into the crystal structure of KLK7 (PDB ID 2QXI), after aligning the protease backbones. Minor clashes were resolved by conjugate-gradients energy minimisation. We then performed molecular dynamic simulations (500 ns, three independent replicates) of this modelled SFTI-TCTR/KLK7 complex, as well as the crystallised SFTI-TCTR/trypsin complex, and an apo-KLK7 structure. For all systems, Cα RMSD values stabilised after the initial 100 ns (S1 Fig), showing a stable binding mode.

**Fig 2:**
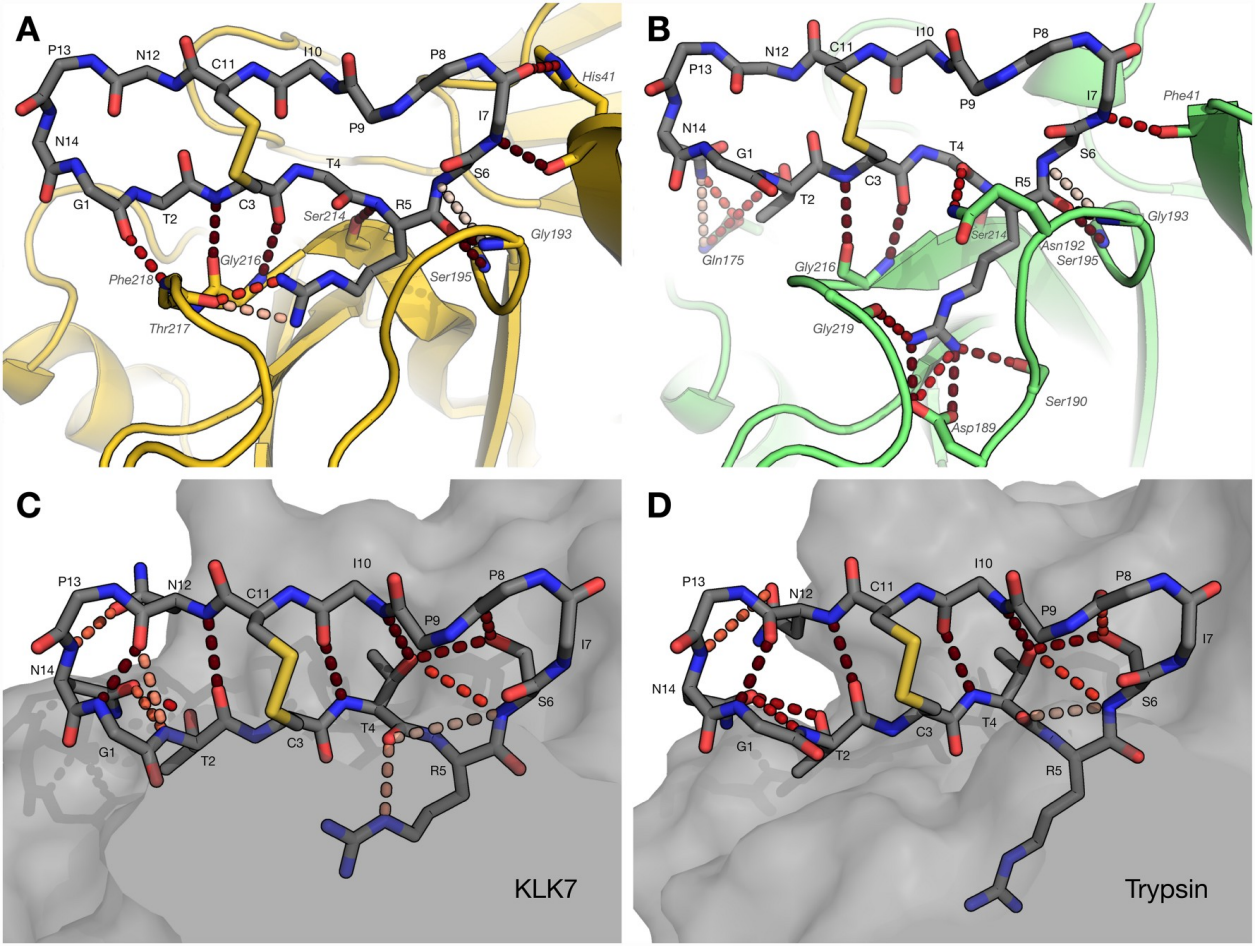
Hydrogen bond analysis of SFTI-TCTR in simulations with KLK7 and trypsin. (A) Intermolecular hydrogen bonds in SFTI-TCTR/KLK7 complex. (B) Intermolecular hydrogen bonds in SFTI-TCTR/trypsin complex. (C) Internal hydrogen bond network of SFTI-TCTR in complex with KLK7. (D) Internal hydrogen bond network of SFTI-TCTR in complex with trypsin. Hydrogen bonds are shown as dashed lines. Occupancy of hydrogen bond is indicated by shade of red: pink (20%) to red-black (>90%). SFTI-TCTR interacts with KLK7 between the P1 Arg_5_ sidechain and Thr_217_ O; all other polar contacts with KLK7 are through its backbone atoms (Gly_1_ O through Ile_7_ O). In contrast, SFTI-TCTR interacts with trypsin using fewer backbone atoms, but more heavily with the S1 pocket through the P1-Arg_5_ sidechain, as well as exploring transient interactions between Gln_175_ and the Asn_14_ sidechain.

**Fig 3:**
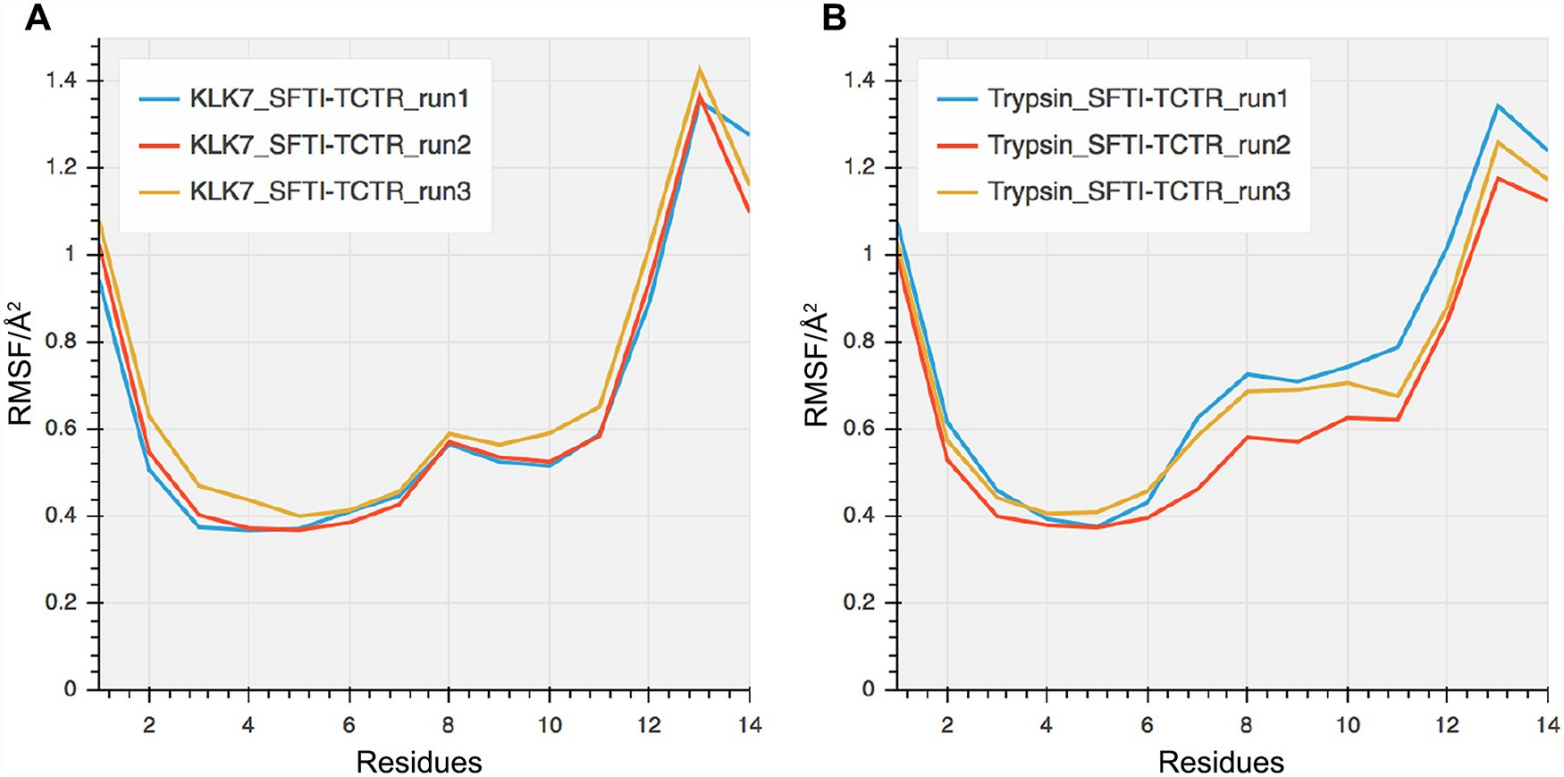
Atomic root mean square fluctuations (RMSF) of inhibitor Cα in three independent MD simulations of (A) the SFTI-TCTR/KLK7 complex, and (B) the SFTI-TCTR/trypsin complex. Residues 7 and 8 show higher levels of order in the KLK7 complex compared to the trypsin complex reflecting the extended backbone interactions from Gly1 to Ile7 as opposed to Cys3 to Ile7 in the trypsin complex.

During the simulations, SFTI-TCTR maintained similar intermolecular interactions with both KLK7 and trypsin between inhibitor residues Cys_3_ N and Arg_5_ O (in the oxyanion pocket), displaying the typical extended β-strand conformation (Fig 2A and 2B). However, SFTI backbone interactions with KLK7 occur over a broader range of well-occupied polar contacts (Gly_1_ O through Ile_7_ O), than with trypsin (Cys_3_ N through Ile_7_ N). The presence of His_41_ in KLK7 (rather than the hydrophobic Phe_41_) explains an observed additional backbone interaction (Ile_7_ O−His_41_ N^δ^). These extended backbone interactions enable SFTI-TCTR to remain more rigid in the context of KLK7 than trypsin (Fig 3), particularly across residues at P2’ to P4’ (_6_IPP_8_).

SFTI-TCTR has a basic P1 residue, Arg_5_, enabling it to strongly engage with the specificity-determining residue Asp_189_ and surrounding backbone atoms in trypsin (Fig 2B). In contrast, KLK7 has a chymotryptic specificity, with specificity-determining residue Asn_189_, and a slightly deeper S1 pocket. The simulation trajectories for the SFTI-TCTR/KLK7 complex reveal that the sidechain of Arg_5_ is only partly introduced to the S1 pocket of KLK7 and does not make contact with the specificity-determining residue Asn_189_ (Fig 2C). Instead, the sidechain of Arg_5_ is deflected away from the base of the S1 pocket forming a high-occupancy hydrogen bond with Thr_217_ carbonyl (Fig 2A). Loop 217–220 of KLK7 is longer than that in trypsin and bulged outwards, widening the entrance of the S1 pocket, and making it possible to accommodate the deflected sidechain of Arg_5_. The aliphatic sidechain of Arg_5_ is well suited to the relatively hydrophobic part of KLK7 S1 pocket near Ala_190_ and Val_213_. The guanidinium group of Arg_5_ forms extra hydrogen bonds with the inhibitor backbone, which may assist in stabilising this conformation (Fig 2C).

The intramolecular H-bond network of SFTI-TCTR remained well-connected, with β-sheet H-bonds Gly_1_ N/Asn_12_ O, Thr_2_ O/Asn_12_ N and Thr_4_ N/Ile_10_ O maintaining >90% occupancy in both simulations. The overall network was similar in both complexes with KLK7 and trypsin (Fig 2C and 2D), though in the presence of KLK7, the P1 Arg_5_ sidechain was able to participate in an additional interaction with Thr_4_ O (Fig 2C). With trypsin, the Thr_2_ sidechain participated in H-bonds with Gln_175_ (loop 6) in trypsin (Fig 2B), resulting in the absence of an internal H-bond between Thr_4_ O^γ^ and Asn_12_ O.

## Discussion

Given the profusion of disease states associated with dysregulated protease activity, administration of exogenous protease inhibitors is an appealing therapeutic strategy (see [50] for a comprehensive review). Whilst there are examples of using unmodified inhibitors such as aprotinin [51] to control aberrant proteolysis, the majority of successful exogenous inhibitor strategies make use of molecules that have been engineered to enhance their potency and selectivity. A recent successful example of this approach is the protease inhibitor drug Kalbitor^®^ used in the treatment of hereditary angioedema [52]. For the most part, modifications are undertaken with the aim of increasing the number or selectivity of contacts between an inhibitor template and its target. This is perhaps somewhat counterintuitive as many naturally occurring inhibitors show relaxed specificity and are able to block the activity of multiple target enzymes. In contrast to these prevailing themes in inhibitor design, we produced a multiple-target SFTI-based inhibitor, SFTI-TCTR, by introducing four mutations, R2T (P4), K5R (P1), F12N, D14N into the cyclic backbone [19]. The design principle behind this variant was to maximise the intramolecular hydrogen bond network while minimising intermolecular sidechain interactions [19]. SFTI-TCTR is not only a highly potent inhibitor of the trypsin-like proteases trypsin, KLK5 and KLK14 but also efficiently blocks the chymotrypsin-like protease KLK7 [19]. Thus, it can control the activities of three pivotal kallikreins in the stratum corneum. Within human skin, kallikreins are controlled by endogenous inhibitor LEKTI with inhibitory *K*_i_s ranging from 3 nM to 300 nM [6]. In the case of LEKTI, effective inhibition against both tryptic and chymotryptic kallikreins is achieved through its multi-domain structure with individual domains targeting a distinctive range of proteases. Thus, a recombinant LEKTI fragment comprising domains 8–11 shows nanomolar *K*_i_s for KLK5 (3.7 nM), KLK7 (34.8 nM) and KLK14 (3.1 nM), while single domain fragments show considerably lower potencies [6]. In contrast, SFTI-TCTR potently inhibits KLK5, KLK7 and KLK14 with a single inhibitory loop. Packaging of this inhibitory potential into a single short peptide sequence is of considerable advantage in terms of therapeutic protease blockade in Netherton Syndrome and atopic dermatitis.

Whilst there are many studies showing inhibition of both tryptic and chymotryptic enzymes by a canonical loop with a P1 arginine residue [53], SFTI-TCTR is unusual in the potency with which it is able to block the chymotrypsin-like activity of KLK7 at nanomolar level. Furthermore, the variant SFTI-TCTR shows a 13-fold increase in KLK7 inhibitory potency compared to SFTI-RCTR, a variant where only the P1 residue of SFTI has been substituted. These two variants have identical P1 residues but show very different side loop properties, indicating the importance of non-contact mutations (R2T, F12N, D12N) in SFTI-TCTR’s potency for KLK7.

Analysis of the crystal structure and simulation trajectories enabled development of an understanding of the interactions and dynamics between the protease active site and the inhibitor. Although the P1 residue Arg_5_ is unfavourable for KLK7 substrate specificity, we predict that its sidechain would be able to adopt a “bent” conformation allowing its accommodation in the S1 pocket of KLK7. In addition to replacing P1 lysine in wildtype SFTI-1 with arginine, residues Arg_2_, Phe_12_ and Asp_14_ were substituted by Thr_2_, Asn_12_ and Asn_14_, respectively. These changes largely minimised the sidechain interactions outside the S1 pocket at the inhibitor/protease interface, while the mainchain interactions and extended β-sheet interactions were maintained in both the SFTI-TCTR/trypsin crystal structure and modelled SFTI-TCTR/KLK7 complex. Molecular simulations showed that the poor S1-P1 contacts resulted from offset binding was compensated by the enhanced mainchain/mainchain interactions across the interface. The fact that these substitutions in SFTI-TCTR resulted in a *K*_i_ of 16.8 nM for KLK7 strongly suggests that the mainchain interactions we describe are sufficient to maintain binding affinity. Interestingly, P1 sidechain distortion is observed in the crystal structures of broad-spectrum inhibitors β-amyloid precursor protein Kunitz domain inhibitor (APPI; PDB ID 1CA0) or BPTI (PDB ID 1CBW) in complexed with chymotrypsin [54]. The P1 sidechains of APPI (P1-Arg) and BPTI (P1-Lys) show a “bent” conformation and donates hydrogen bonds to the carbonyl of Thr_217_ as well as to the backbone of inhibitor molecule. The P1 Arg/Lys interacts with the S1 subsite of chymotrypsin-like proteases in a shallower manner compared to that of trypsin-like proteases. This may represent a common conformation when P1 Arg/Lys residues are accommodated by the S1 pocket of chymotrypsin-like proteases.

From a developmental perspective, the evolution of protease inhibitors must parallel that of their target proteases. In the human degradome, 570 proteases have been identified, whose activities are controlled by about 156 protease inhibitors [55]. Thus there is a trend toward developing highly “efficient” inhibitors which bind to multiple target proteases. The structural driving force behind this phenomenon is likely to be that the mainchain conformation of serine proteases is more constrained than sidechain position within the wider serine protease family. Inhibitors forming strong hydrogen bonds with the mainchain atoms of the protease active site are more likely to maintain the same interactions within homologous proteases. This affords an important molecular design opportunity in that new inhibitors could be constructed to target the mainchain motif of these proteases rather than sidechain interactions, providing a simple protein engineering solution to providing potent, broad-range, serine protease inhibition.

## Supporting information

S1 Fig Cα RMSD of molecular dynamic simulations. After a least-squares fit on Cα to the initial frame, the Cα RMSD for each frame in the trajectories was calculated, smoothed with a 1 ns Savitzky-Golay filter, and plotted. All systems plateau after ~100ns between 3−3.5 Å.
(TIF)

S2 Fig Cα RMSF of molecular dynamic simulations. (A) individual *apo*KLK7 runs, (B) individual SFTI-TCTR/KLK7 runs, (C) individual SFTI-TCTR/trypsin runs, and (D) overall RMSF of each system. Residue numbering follows chymotrypsin numbering, with trypsin in yellow where it differs from KLK7. The presence of SFTI-TCTR reduced the flexibility of KLK7 in simulation, as is expected for an inhibitor. The SFTI-TCTR/trypsin complex however, was the most mobile of all systems simulated, particularly around loop 3.
(TIF)

S1 File Crystal structure of trypsin/SFTI-TCTR complex (PDB ID 6BVH). (ZIP)
