# Supplementary figures and images for: Potent, multi-target serine protease inhibition achieved by a simplified β-sheet motif
Source: PLoS One. 2019 Jan 22;14(1):e0210842. doi: 10.1371/journal.pone.0210842 (PMC6342301; doi:10.1371/journal.pone.0210842)

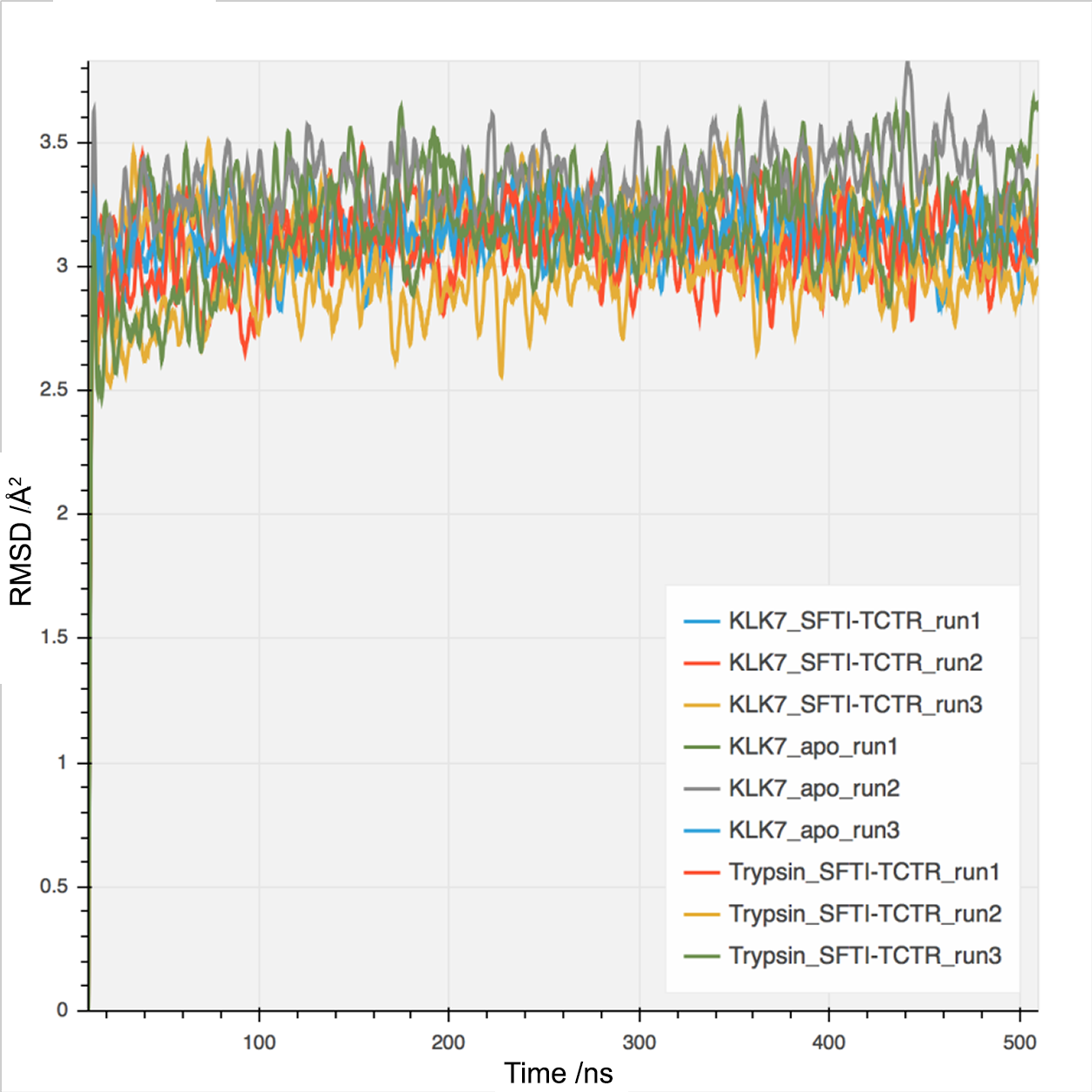

Supplement: S1 Fig — After a least-squares fit on Cα to the initial frame, the Cα RMSD for each frame in the trajectories was calculated, smoothed with a 1 ns Savitzky-Golay filter, and plotted. All systems plateau after ~100ns between 3−3.5 Å. (TIF) [file pone.0210842.s001.tif]

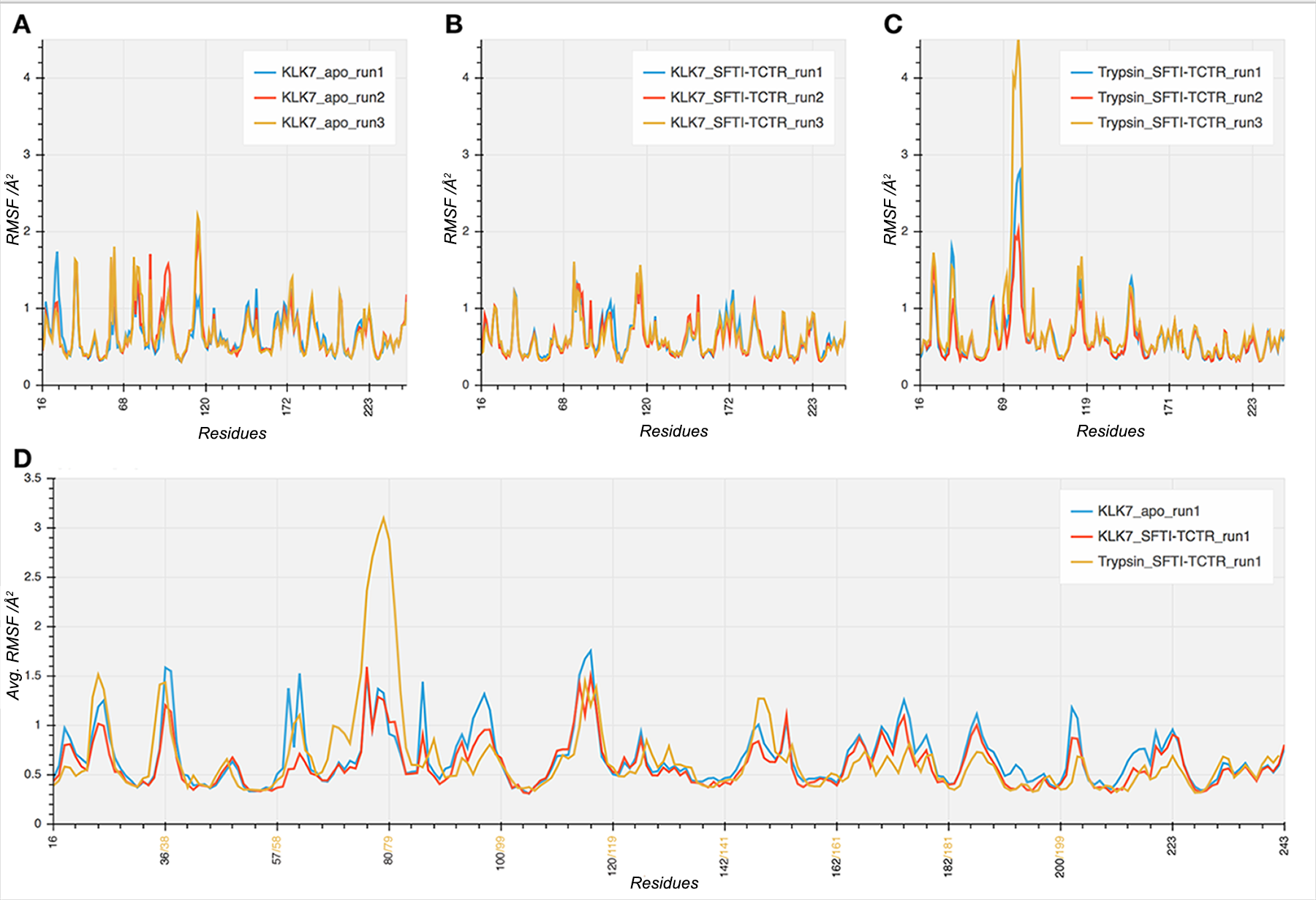

Supplement: S2 Fig — (A) individual apoKLK7 runs, (B) individual SFTI-TCTR/KLK7 runs, (C) individual SFTI-TCTR/trypsin runs, and (D) overall RMSF of each system. Residue numbering follows chymotrypsin numbering, with trypsin in yellow where it differs from KLK7. The presence of SFTI-TCTR reduced the flexibility of KLK7 in simulation, as is expected for an inhibitor. The SFTI-TCTR/trypsin complex however, was the most mobile of all systems simulated, particularly around loop 3. (TIF) [file pone.0210842.s002.tif]
